# Contribution of Matrix Metalloproteinase-2 and Matrix Metalloproteinase-9 to Upper Tract Urothelial Cancer Risk in Taiwan

**DOI:** 10.3390/life14070801

**Published:** 2024-06-26

**Authors:** Bo-Ren Wang, Hung-Huan Ma, Chao-Hsiang Chang, Cheng-Hsi Liao, Wen-Shin Chang, Mei-Chin Mong, Ya-Chen Yang, Jian Gu, Da-Tian Bau, Chia-Wen Tsai

**Affiliations:** 1 Graduate Institute of Biomedical Sciences, China Medical University, Taichung 404333, Taiwan; 2 Division of Urology, Department of Surgery, Taichung Armed Forces General Hospital, Taichung 41152, Taiwan; 3 National Defense Medical Center, Taipei 11490, Taiwan; 4 Division of Nephrology, Department of Internal Medicine, Taichung Tzu Chi Hospital, Taichung 427003, Taiwan; 5 Terry Fox Cancer Research Laboratory, Department of Medical Research, China Medical University Hospital, Taichung 404327, Taiwan; 6 Department of Urology, China Medical University Hospital, Taichung 404327, Taiwan; 7 Department of Epidemiology, The University of Texas MD Anderson Cancer Center, Houston, TX 77030, USA; 8 Department of Food Nutrition and Health Biotechnology, Asia University, Taichung 413305, Taiwan; 9 Department of Bioinformatics and Medical Engineering, Asia University, Taichung 413305, Taiwan

**Keywords:** genotype, phenotype, MMP-2, MMP-9, polymorphism, upper tract urothelial cancer

## Abstract

Matrix metalloproteinase (MMP)-2 and -9, which degrade type IV collagen, are linked to cancer invasion and metastasis. Gene polymorphisms in *MMP-2* and *MMP-9* can influence their function, impacting cancer development and progression. This study analyzed the association between polymorphisms *MMP-2* rs243865 (C-1306T), rs2285053 (C-735T), and *MMP-9* rs3918242 (C-1562T) with serum concentrations of these enzymes in upper tract urothelial cancer (UTUC) patients. We conducted a case–control study with 218 UTUC patients and 580 healthy individuals in Taiwan. Genotyping was performed using PCR/RFLP on DNA from blood samples, and MMP-2 and MMP-9 serum levels and mRNA expressions in 30 UTUC patients were measured using ELISA and real-time PCR. Statistical analysis showed that *MMP-2* rs2285053 and *MMP-9* rs3918242 genotypes were differently distributed between UTUC patients and controls (*p* = 0.0199 and 0.0020). The *MMP-2* rs2285053 TT genotype was associated with higher UTUC risk compared to the CC genotype (OR = 2.20, *p* = 0.0190). Similarly, *MMP-9* rs3918242 CT and TT genotypes were linked to increased UTUC risk (OR = 1.51 and 2.92, *p* = 0.0272 and 0.0054). In UTUC patients, TT carriers of *MMP-2* rs2285053 and *MMP-9* rs3918242 showed higher mRNA and protein levels (*p* < 0.01). These findings suggest that *MMP-2* rs2285053 and *MMP-9* rs3918242 genotypes are significant markers for UTUC risk and metastasis in Taiwan.

## 1. Introduction

Urothelial carcinoma, one of the most prevalent tumors, can originate in the upper urinary tract (pyelocaliceal cavities and ureter) or lower urinary tract (bladder and urethra). Upper tract urothelial carcinoma (UTUC) is specifically referred to when the carcinoma arises in the upper tract and is often studied separately. In Taiwan, the incidence of urothelial tumors has been steadily increasing over recent years [1]. While UTUC accounts for only 5% of urothelial carcinomas in Western countries [2,3], it constitutes up to 31% in Taiwan [4,5,6], making Taiwan a high-incidence area for UTUC [7]. This discrepancy is possibly linked to environmental risk factors, such as arsenic-contaminated drinking water [8] and aristolochic acid from Chinese herbal medicine [9]. Given the higher UTUC incidence in Taiwan, genomic and proteomic studies in this population could yield valuable insights when compared to Western populations. Although risk factors such as smoking [10], analgesic abuse [11], arsenic contamination [12], and occupational exposure [9] have been associated with UTUC, these have provided limited clinical utility. Recent evidence suggests that genetic variations may predispose individuals to UTUC, potentially serving as predictive indicators [13,14].

Matrix metalloproteinases (MMPs), also known as matrixins, are a group of peptidases integral to inflammation, carcinogenesis, and cancer cell migration through the regulation of extracellular matrix (ECM) components [15,16]. Among these, MMP-2 and MMP-9 are particularly well studied. As gelatinases, MMP-2 and MMP-9 degrade type IV collagen in the basement membrane, contributing to carcinogenic processes such as cell proliferation, angiogenesis, and tumor metastasis when their activity is dysregulated [17,18,19]. These enzymes are crucial for maintaining cellular processes, including growth, inflammation, wound healing, cell remodeling, and angiogenesis. Research indicates that MMP-2 and MMP-9 play significant roles in cancer cell proliferation and invasion, impacting tumor growth, aggressive progression, and patient survival in UTUC [20,21].

In recent years, numerous genetic studies have explored the associations between *MMP-2* and *MMP-9* polymorphisms and cancer risk across various types of cancer, including head and neck [22,23,24], lung [25,26,27,28], esophageal [29,30,31], breast [32,33,34,35], hepatocellular [36,37], gastric [30,38,39,40], colorectal [41,42,43,44], gallbladder [45,46], cervical [47], bladder [48,49], renal [50], and prostate cancer [51,52,53] in diverse populations. However, no studies have been published on the role of *MMP-2* or *MMP-9* genotypes in UTUC patients indexed in the MEDLINE (PubMed) database. This gap may be due to the low incidence of UTUC, challenges in sample collection, and limited sample sizes.

Given this context, our study aims to investigate the potential association between *MMP-2* rs243865 (C-1306T), rs2285053 (C-735T), and *MMP-9* rs3918242 (C-1562T) genotypes and the risk of developing UTUC in a Taiwanese cohort of 218 UTUC patients and 580 healthy controls. Additionally, we will explore the genotype–phenotype correlation of *MMP-2* and *MMP-9* and assess the metastatic potential related to these MMPs to understand the aggressiveness of UTUC.

## 2. Materials and Methods

### 2.1. Recruitment of UTUC Patients and Non-UTUC Control Groups

A total of 218 UTUC patients were recruited at China Medical University, all diagnosed via pathological examination of biopsy or surgical resection specimens. Clinical and histopathological data were meticulously collected from patient charts and pathological reports, subsequently reviewed, and entered into a database. Tumor staging was conducted using the TNM system [54], while pathological grading followed the World Health Organization criteria [55]. For the control group, 580 healthy individuals, matched by age and admitted to the same hospital for health checkups, were selected. These controls had no history of neoplastic urological disease or other malignancies. All participants provided informed consent, and the study was approved by the Human Research Committees (DMR104-IRB-158).

### 2.2. Genotyping Methodology of MMP-2 rs243865 and rs2285053

In this study, DNA was extracted from the peripheral blood leukocytes of each participant using the QIAamp Blood Mini Kit (Blossom, Taipei, Taiwan), following the methodology outlined in previous publications [56,57,58]. The primers, restriction endonucleases, and PCR conditions for genotyping *MMP-2* rs243865 and rs2285053 were consistent with our earlier publications [51,59]. Specifically, PCR fragments were digested overnight with restriction enzymes *Xsp* I and *Hinf* I (New England Biolabs, Taipei, Taiwan) for *MMP-2* rs243865 and rs2285053, respectively. The genotyping profiles were subsequently analyzed by two independent researchers using 3% agarose gel electrophoresis to ensure accuracy.

### 2.3. Genotyping Methodology of MMP-9 rs3918242

The primer design, selection of restriction endonucleases, and PCR conditions for genotyping *MMP-9* rs3918242 followed the protocols established in our previous studies [60,61]. PCR fragments were digested overnight with the restriction enzyme *Sph* I (New England Biolabs, Taipei, Taiwan) for *MMP-9* rs3918242. The genotyping results, based on the digestibility of C allele and T allele DNA sequences with *Sph* I, were as follows: the CC genotype produced a 386 bp fragment, the CT genotype produced fragments of 386, 320, and 66 bp, and the TT genotype produced fragments of 320 and 66 bp.

### 2.4. Transcriptional Expression of MMP-2 and MMP-9

To investigate the relationship between mRNA expression and high-risk *MMP-2* and *MMP-9* genotypes, we collected 30 tissue samples from UTUC patients and extracted RNA using Qiagen RNA extraction kits. Real-time quantitative PCR was performed using an FTC-3000 instrument (Funglyn Biotech Inc., Toronto, ON, Canada). Glyceraldehyde 3-phosphate dehydrogenase (GAPDH) served as the internal control for quantitative analysis, as previously described [62,63]. The primer sequences were as follows: for *MMP-2* mRNA, forward 5′-GTGCTTACCTAGCACATGCAAT-3′ and reverse 5′-CGCATGGTCTCGATGGTATTC-3′; for *MMP-9* mRNA, forward 5′-TTCCTTGGTCTGGTGTCCC-3′ and reverse 5′-CCCACTTCTTGTCGCTGTC-3′; and for GAPDH mRNA, forward 5′-GAAATCCCATCACCATCTTCCAGG-3′ and reverse 5′-GAGCCCCAGCCTTCTCCATG-3′. The results were averaged from three independent tests and normalized to GAPDH expression.

### 2.5. Translational Expression of MMP-2 and MMP-9

Protein extraction from the 30 UTUC patient tissue samples was conducted as described in previous publications [64,65]. Protein concentrations were determined using the Bradford protein assay reagent (Bio-Rad, Hercules, CA, USA) with BSA as the standard. Protein extracts were prepared in sample buffer (62 mM Tris–HCl, 2% SDS, 10% glycerol, 5% β-mercaptoethanol) and heated at 97 °C for 5 min. Equal amounts (50 µg) of denatured protein were loaded per lane, separated by 8–15% SDS-PAGE, and transferred onto PVDF membranes overnight. Membranes were blocked with 5% non-fat dried milk in PBS containing 1% Tween-20 for 1 h at room temperature, then incubated with primary antibodies against MMP-2 and MMP-9 for 2 h. Subsequently, blots were incubated with HRP-conjugated anti-mouse or anti-rabbit secondary antibodies (1:5000) overnight at room temperature. Detection was performed using the Immobilon Western-HRP Substrate (Millipore, Billerica, MA, USA) with enhanced chemiluminescence.

### 2.6. Statistical Analysis Methodology

To ensure the control group accurately represented the general population, a Hardy–Weinberg equilibrium assessment was conducted using a goodness-of-fit test to detect deviations in genotype frequencies at polymorphic sites on the *MMP-2* and *MMP-9* genes. An unpaired Student’s *t*-test was used to compare various parameters, including age and quantitative mRNA and protein levels, between the case and control groups. Pearson’s chi-square test with Yates’ correction was utilized to compare the distribution of genotypes among subgroups. A *p*-value of less than 0.05 was considered statistically significant for all tests. Logistic regression analysis was employed to estimate odds ratios (ORs) and 95% confidence intervals (CIs) for genotypes associated with UTUC.

## 3. Results

### 3.1. Demographic Characteristics of the UTUC Population

Table 1 displays the frequency distributions of clinical characteristics among participants, including 218 UTUC patients and 580 healthy controls. Epidemiologically, gender (*p* = 0.4256) and age (*p* = 0.8518) showed no significant differences, indicating well-matched populations. From a clinical and pathological perspective, tumors were distributed across renal pelvic (38.5%), ureter (34.9%), and multiple sites (26.6%). Among UTUC patients, 18.8% experienced metastasis, 60.6% had high-grade tumors, and 77.1% were at stages lower than pT3 (Table 1).

### 3.2. The Genotyping Outcomes for the UTUC Patients and Non-UTUC Control Groups

Table 2 illustrates the distribution of *MMP-2* rs243865, rs2285053, and *MMP-9* rs3918242 genotypes among 218 UTUC cases and 580 non-UTUC healthy controls. The analysis revealed differential distribution of *MMP-2* rs2285053 and *MMP-9* rs3918242 genotypes between the UTUC and non-UTUC control groups (*p* for trend = 0.0199 and 0.0020, respectively). Specifically, the *MMP-2* rs2285053 homozygous variant TT was associated with increased UTUC risk compared to the wild-type CC genotype (OR = 2.20, 95%CI = 1.18–4.10, *p* = 0.0190). However, the *MMP-2* rs2285053 heterozygous variant CT did not show association with UTUC risk (OR = 1.34, 95%CI = 0.97–1.87, *p* = 0.0946). In the dominant model, *MMP-2* rs2285053 CT + TT genotypes were significantly higher in the UTUC group compared to the non-UTUC control group (OR = 1.44, 95%CI = 1.05–1.98, *p* = 0.0261). Regarding *MMP-9* rs3918242, both the heterozygous variant CT and homozygous variant TT were associated with elevated UTUC risk compared to the wild-type CC genotype (OR = 1.51 and 2.92, 95%CI = 1.06–2.15 and 1.41–6.06, *p* = 0.0272 and 0.0054, respectively). The *MMP-9* rs3918242 CT + TT genotypes were significantly higher in the UTUC group compared to the non-UTUC control group (OR = 1.66, 95%CI = 1.19–2.32, *p* = 0.0035). Conversely, none of the genetic models showed *MMP-2* rs243865 to be associated with UTUC risk.

### 3.3. The Allelic Frequency Distribution Analyzing Outcomes for the UTUC Patients and Non-UTUC Control Groups

In the UTUC group, the frequency of the *MMP-2* rs243865 variant allele T was 11.5%, which did not significantly differ from the 10.1% observed in the non-UTUC control group (OR = 1.15, 95%CI = 0.81–1.64, *p* = 0.4766, Table 3 top part). Consistent with the findings in Table 2, the allelic frequencies of the variant T alleles for *MMP-2* rs2285053 and *MMP-9* rs3918242 were both significantly higher in UTUC cases than in the non-UTUC control groups (Table 3 middle and bottom parts).

### 3.4. The mRNA and Protein Expression Levels of MMP-2 and MMP-9

**Figure 1:**
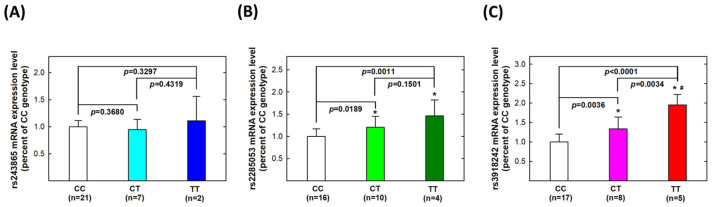
Expression levels of mRNA transcripts of *MMP-2* and *MMP-9* genes in tissue samples collected from patients with UTUC according to genotype at *MMP-2* rs243865 (**A**), *MMP-2* rs2285053 (**B**), and *MMP-9* rs3918242 (**C**). The average (fold) expression levels were normalized, applying GAPDH as an internal standard. Each assay was conducted at least three times. * Significantly different from CC genotypes; # significantly different from CT genotypes.

We aimed to explore the correlations between *MMP-2* and *MMP-9* genotypes and their phenotypes. Firstly, we analyzed genotype-based mRNA expression levels among 30 UTUC patients. At the *MMP-2* rs243865 polymorphic site, twenty-one, seven, and two patients were CC, CT, and TT carriers, respectively. No statistically significant difference was observed in *MMP-2* mRNA expression levels among CC, CT, or TT carriers (all *p* > 0.05, Figure 1A). For *MMP-2* rs2285053, 16, 10, and 4 patients carried the CC, CT, and TT genotypes. The mRNA transcript level was significantly higher in CT carriers (*p* = 0.0189) and even higher in TT carriers (*p* = 0.0011) compared to CC carriers (Figure 1B). Regarding *MMP-9* rs3918242, CT carriers exhibited higher mRNA transcript levels than CC carriers (*p* = 0.0036), while TT carriers had higher levels than both CT (*p* = 0.0034) and CC (*p* < 0.0001) carriers (Figure 1C).

**Figure 2:**
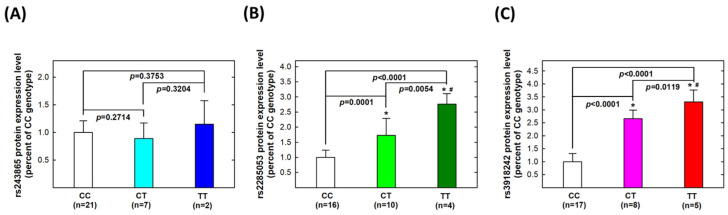
Expression levels of proteins of *MMP-2* and *MMP-9* genes in tissue samples collected from patients with UTUC according to genotype at *MMP-2* rs243865 (**A**), *MMP-2* rs2285053 (**B**), and *MMP-9* rs3918242 (**C**). The average (fold) expression levels were normalized, applying β-actin as an internal standard. Each assay was conducted at least three times. * Significantly different from CC genotypes; # significantly different from CT genotypes.

Secondly, we investigated genotype-based protein levels among the same 30 UTUC patients. No statistically significant difference was found in MMP-2 protein expression levels among CC, CT, or TT carriers of *MMP-2* rs243865 (all *p* > 0.05, Figure 2A). For *MMP-2* rs2285053, protein expression levels were significantly higher in CT carriers (*p* = 0.0001) and further higher in TT carriers (*p* = 0.0054) compared to CC carriers (Figure 2B). Regarding *MMP-9* rs3918242, CT carriers exhibited higher protein expression levels than CC carriers (*p* < 0.0001), while TT carriers had higher levels than both CT (*p* = 0.0119) and CC (*p* < 0.0001) carriers (Figure 2C).

### 3.5. The Associations of SNPs with the Risks of Metastasis in UTUC Patients

Given the reported predictive role of MMP-2 and/or MMP-9 overexpression in metastatic progression in various cancers, we sought to investigate whether *MMP-2* and/or *MMP-9* genotypes were linked to metastatic risk in UTUC. We found that patients carrying the variant genotypes (CT + TT) of *MMP-2* rs2285053 and *MMP-9* rs3918242 were associated with significantly increased risks of metastasis (OR = 2.47, 95% CI = 1.20–4.98; and OR = 3.47, 95% CI = 1.71–7.00, respectively). *MMP-2* rs243865 showed no significant association (Table 4).

## 4. Discussion

Over the past decade, the Cancer Genome Atlas (TCGA) has been instrumental in linking molecular biomarkers with comprehensive insights into the molecular pathways underlying carcinogenesis, tumor progression, and potential therapeutic targets [66]. Despite this, studies on molecular markers for UTUC have predominantly focused on tissue-based markers like p53 [67], Ki67 [68], and HER-2 [69], often limited by sample size. As early as 2000, Nakanish et al. reported a positive correlation between MMP-2 staining levels and UTUC stage in 102 Japanese cases [70]. Subsequent studies by Miyata et al. provided evidence of MMP-2 expression significantly correlating with tumor stage and grade in 91 UTUC patients, although predictive impacts on survival were insignificant [71]. In 2005, Kamijima et al. found little correlation between p53, Ki-67, MMP-2, and MMP-9 overexpression and survival status among 69 UTUC patients, except for Ki-67 [72]. However, invasive sampling inconveniences and errors from tissue staining hinder precision medicine. In our case–control study, we examined *MMP-2* and *MMP-9* genotypic profiles in 798 Taiwanese subjects, including 218 UTUC cases and 580 non-UTUC controls (as shown in Table 1). Our study, the first to assess *MMP-2* and *MMP-9* genotypes’ impact on UTUC risk, highlighted the differential distribution of *MMP-2* rs2285053 and *MMP-9* rs3918242 genotypes between UTUC and control groups (in Table 2). This supports the potential of *MMP-2* rs2285053 and *MMP-9* rs3918242 variant genotypes as novel diagnostic predictors for UTUC, with *MMP-9* rs3918242 possibly more sensitive, as even the heterozygous variant genotype reached significance (Table 2).

We conducted a genotype–phenotype correlation analysis on tumor tissues from thirty UTUC patients. Our findings revealed significantly higher mRNA transcript and protein levels in variant genotype carriers compared to wild-type genotype carriers for both *MMP-2* rs2285053 and *MMP-9* rs3918242 polymorphic sites (Figure 1 and Figure 2). Despite studies by Nakanish, Miyata, and Kamijima’s groups not supporting *MMP-2* rs2285053 or *MMP-9* rs3918242 as effective overall survival predictors [70,71,72], our results provide evidence of their significant roles in determining and predicting metastatic potential (Table 3). The presence of the variant T allele at both *MMP-2* rs2285053 and *MMP-9* rs3918242 appeared to elevate metastatic risk. Importantly, MMP-2 and MMP-9 emerged as determinants and predictors for UTUC, not only in diagnostic susceptibility but also prognostic metastatic potential. In the existing literature, the only MMP member reported to associate with metastasis is MMP-11. In 2016, MMP-11 overexpression was associated with aggressive tumor phenotype and unfavorable clinical outcomes in UTUC [73].

We hypothesized that MMPs play a pivotal role in ECM remodeling and are implicated in various processes of carcinogenesis. The clinical significance of MMPs, particularly MMP-2 and MMP-9, has been elucidated in numerous dysregulated conditions such as neoplastic, autoimmune, and chronic inflammation disorders [74,75,76]. MMP-mediated degradation of ECM components facilitates tumor cell invasion and metastasis in the microenvironment [77,78]. Among MMP members, MMP-1, -2, -3, -9, and -13 have been exclusively associated with cancer metastasis [79,80]. In the literature, MMP-2 and MMP-9 are frequently implicated in the development and expansion of tumor cells in bone metastasis [81,82,83]. In our study, we found that the *MMP-2* rs2285053 and *MMP-9* rs3918242 genotypes were associated with metastasis (Table 4). Risky genotypes (CT and TT) of *MMP-2* rs2285053 and *MMP-9* rs3918242 correlated with elevated transcriptional and translational levels of MMP-2 and MMP-9 (Figure 1 and Figure 2). MMP-9 and MMP-2 belong to gelatinases, one of the five groups in the MMP family, based on structure and substrate specificity [84]. MMP-2 specifically degrades type IV collagen and denatured collagens [85]. Our genotype-based phenotypic results showed that mRNA and protein expression of MMP-2 were highest in TT carriers, followed by CT and CC carriers at both *MMP-2* rs2285053 and *MMP-9* rs3918242 sites (Figure 1 and Figure 2). MMP-9 enhances tumor cell metastatic capacity by degrading collagen proteins of the ECM after activation by extracellular proteases [86,87]. In our study, mRNA and protein levels of MMP-9 were highest in TT carriers, followed by CT carriers, then CC carriers at both *MMP-2* rs2285053 and *MMP-9* rs3918242 (Figure 1 and Figure 2). Notably, numerous studies have demonstrated that MMP-2 and MMP-9 inhibition undermines tumor metastasis capability [88,89,90,91]. Several research groups have proposed strategies for enhancing MMP inhibitors’ effectiveness, particularly MMP-2 and MMP-9, in cancer treatments [92,93]. Further investigation is warranted to ascertain whether MMP-2 and/or MMP-9 are significantly more highly expressed in UTUC tissues compared to adjacent non-UTUC tissues and if the suppression of MMP-2 and/or MMP-9 can reduce the metastatic capacity of UTUC primarily cultured cells.

Although we have shown that SNPs in MMP2 and MMP9 are associated with increased risks of UTUC and metastasis, the effect of individual SNPs on cancer risks and outcomes is modest and not clinically applicable. Recent studies have shown the potential clinical utility of polygenic risk scores using multiple SNPs in increasing predictive power [94,95]. Furthermore, epigenetic modifications, most notably, DNA methylation, are key players in driving cancer development and have shown great potential as biomarkers for cancer risk prediction, early detection, and prognosis [96,97,98]. Future studies should incorporate genetic, epigenetic, environmental, and clinical factors to build comprehensive models for risk stratification and outcome prediction.

**Figure 3:**
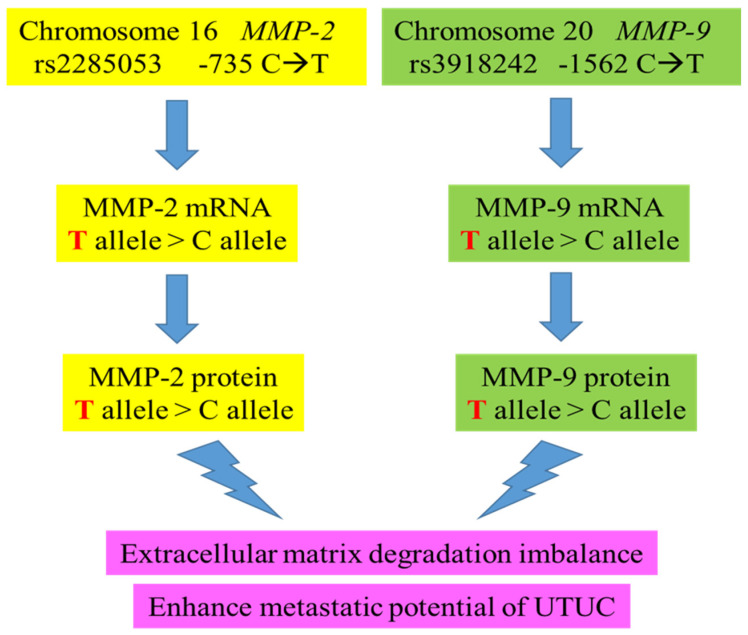
The proposed genetic influence of *MMP-2* and *MMP-9* genotypes on their mRNAs, protein levels, and UTUC clinical endpoints of this study.

We have encapsulated the entire study into an easily understandable summary (Figure 3). In this investigation, we examined three SNPs known for their functional impact: rs243865 and rs2285053 of the *MMP-2* gene in its promoter region and rs3918242 of the *MMP-9* gene at position -1562. The rs2285053 SNP of *MMP-2*, situated at position -735, entails a C-to-T transition known to disrupt the binding site of specificity protein 1 (Sp1) to its mRNA, resulting in reduced transcription levels [99]. Similarly, rs3918242 of the *MMP-9* gene at position -1562 also alters its promoter activity [28]. The genotypes of *MMP-2* rs2285053 and *MMP-9* rs3918242 exhibited strong correlations with the mRNA levels of MMP-2 and MMP-9, suggesting that specific variant genotypes might influence the concentrations of both MMP-2 and MMP-9 proteins. This could potentially elevate the risk of UTUC and enhance its metastatic potential, thereby contributing to a more aggressive UTUC phenotype (Figure 3).

There are a few limitations to this study. First, this is a single-center study in Taiwan. Multi-center studies in diverse populations can increase the generalizability of our findings to other populations. Second, while the overall sample size was large, the number of tissue samples was limited, which may impact the robustness of the mRNA and protein expression analyses. Third, the clinical utility of SNPs remains limited. Genetic, epigenetic, environmental, and clinical factors are all important for risk prediction. Fourth, this study is based on a case–control design, which limits the ability to establish causality or track the progression of UTUC over time. Fifth, this study controls for some demographic variables but may not account for all the potential confounding factors that could influence UTUC risk and progression. Future studies should address these limitations.

## 5. Conclusions

In conclusion, our pilot study suggests that *MMP-2* rs2285053 and *MMP-9* rs3918242 hold promise as pioneering diagnostic indicators for early UTUC detection, supported by compelling phenotypic evidence. Furthermore, their potential as prognostic markers for UTUC metastatic potential adds significant value to their clinical relevance. Urgent efforts are warranted for further investigations across diverse geographical regions to elucidate the intricate involvement of MMPs, particularly MMP-2 and MMP-9, in UTUC etiology.

## Figures and Tables

**Table 1:** Demographics of the 218 UTUC patients and 580 healthy controls.

Characteristics	Cases (*n* = 218)		Controls (*n* = 580)		*p*-Value
	N	%	N	%	
Age (mean ± SD)	65.4 ± 4.7		62.9 ± 3.9		0.8518 ^a^
Gender					
Male	114	52.3%	323	55.7%	0.4256 ^b^
Female	104	47.7%	257	44.3%	
Location					
Renal pelvic tumor	84	38.5%			
Ureter tumor	76	34.9%			
Multiple tumor	58	26.6%			
Lymph node metastasis					
Yes	41	18.8%			
No	177	81.2%			
Grade					
Low	86	39.4%			
High	132	60.6%			
Stage					
I or II	168	77.1%			
III or IV	50	22.9%			

Statistical analysis was based on ^a^ unpaired Student’s *t*-test and ^b^ Pearson’s chi-square with Yates’ correction test.

**Table 2:** Genotypic frequency distributions of *MMP-2* rs243865, rs2285053, and *MMP-9* rs3918242 among 218 UTUC patients and 580 healthy controls.

Genotypes	Controls, *n* (%)	Cases, *n* (%)	OR (95%CI)	*p*-Value ^a^
*MMP-2* promoter -1306				
rs243865				
CC	472 (81.4)	173 (79.4)	1.00 (Reference)	
CT	99 (17.1)	40 (18.3)	1.10 (0.73–1.66)	0.7152
TT	9 (1.5)	5 (2.3)	1.52 (0.50–4.59)	0.6620
CT + TT	108 (18.6)	45 (20.6)	1.14 (0.77–1.68)	0.5854
*P*_trend_				0.6955
*P*_HWE_				0.1559
*MMP-2* promoter -735				
rs2285053				
CC	353 (60.9)	113 (51.8)	1.00 (Reference)	
CT	200 (34.5)	86 (39.5)	1.34 (0.97–1.87)	0.0946
TT	27 (4.6)	19 (8.7)	**2.20 (1.18–4.10)**	0.0190 *
CT + TT	227 (39.1)	105 (48.2)	**1.44 (1.05–1.98)**	0.0261 *
*P*_trend_				0.0199 *
*P*_HWE_				0.8444
*MMP-9* promoter -1562				
rs3918242				
CC	430 (74.1)	138 (63.3)	1.00 (Reference)	
CT	134 (23.1)	65 (29.8)	**1.51 (1.06–2.15)**	0.0272 *
TT	16 (2.8)	15 (6.9)	**2.92 (1.41–6.06)**	0.0054 *
CT + TT	150 (25.9)	80 (36.7)	**1.66 (1.19–2.32)**	0.0035 *
*P*_trend_				0.0020 *
*P*_HWE_				0.1627

OR: odds ratio; CI: confidence interval; ^a^ data based on chi-square test with Yates’ correction; *P*_trend_: *p*-Value based on trend analysis; *P*_HWE_: *p*-Value based on Hardy–Weinberg equilibrium; * significant data and bolded.

**Table 3:** Allelic frequencies for *MMP-2* rs243865, rs2285053, and *MMP-9* rs3918242 among 218 UTUC patients and 580 healthy controls.

Allelic Types	Controls, *n* (%)	Cases, *n* (%)	OR (95%CI)	*p*-Value ^a^
*MMP-2* rs243865				
Allele C	1043 (89.9)	386 (88.5)	1.00 (Reference)	
Allele T	117 (10.1)	50 (11.5)	1.15 (0.81–1.64)	0.4766
*MMP-2* rs2285053				
Allele C	906 (78.1)	312 (71.6)	1.00 (Reference)	
Allele T	254 (21.9)	124 (28.4)	**1.42 (1.10–1.82)**	0.0075 *
*MMP-9* rs3918242				
Allele C	944 (85.7)	341 (78.2)	1.00 (Reference)	
Allele T	166 (14.3)	95 (21.8)	**1.58 (1.20–2.10)**	0.0016 *

OR: odds ratio; CI: confidence internal; ^a^ data based on chi-square test with Yates’ correction; * significant data and bolded.

**Table 4:** Associations of *MMP-2* rs243865, rs2285053, and *MMP-9* rs3918242 with the risks of metastasis among 218 UTUC patients.

Genotypes	Metastasis		OR (95%CI)	*p*-Value ^a^
	No	Yes		
*MMP-2* rs243865				
CC	142	31	1.00 (Reference)	0.6571
CT + TT	35	10	1.31 (0.59–2.92)	
*MMP-2* rs2285053				
CC	99	14	1.00 (Reference)	0.0192 *
CT + TT	78	27	**2.47 (1.20–4.98)**	
*MMP-9* rs3918242				
CC	122	16	1.00 (Reference)	0.0007 *
CT + TT	55	25	**3.47 (1.71–7.00)**	

OR: odds ratio; CI: confidence internal; ^a^ data based on chi-square test with Yates’ correction; * significant data and bolded.

## Data Availability

The genotyping results and clinical data supporting the findings of this study are available from the corresponding author upon reasonable requests via email at 017891@tool.caaumed.org.tw.
